# Measuring the Quality of Life in Forensic Psychiatric Hospitals

**DOI:** 10.3389/fpsyg.2021.701231

**Published:** 2021-07-09

**Authors:** Michael Büsselmann, Larissa Titze, Maximilian Lutz, Manuela Dudeck, Judith Streb

**Affiliations:** Department of Forensic Psychiatry and Psychotherapy, Ulm University, Ulm, Germany

**Keywords:** quality of life, well-being, forensic psychiatry, mentally ill offenders, living conditions

## Abstract

**Background:** In Germany, a large proportion of mentally ill offenders spends many years in a forensic psychiatric hospital. To ensure that the highly restrictive living conditions in these closed institutions meet patient needs, research must assess and analyze patient quality of life. For this purpose, we adapted the Measuring the Quality of Prison Life questionnaire to measure the quality of life in forensic psychiatric hospitals from the patient perspective. This study aimed to assess the reliability (internal consistency) and construct validity of the adapted questionnaire.

**Methods:** To evaluate the questionnaire, a one-time survey was carried out at 13 forensic psychiatric hospitals in Germany. Item characteristics and internal consistency of the scale and subscales were calculated and the factor structure was tested using confirmatory factor analysis. To test of responsiveness we compared the mean quality of life between the 13 hospitals and further investigated whether the patients' evaluation of quality of life is depending on age and duration of accommodation.

**Results:** The analysis of the psychometric properties revealed very good item characteristics and very good to excellent internal reliability. Construct validity was demonstrated. Patient's quality of life was significantly associated with age and duration of accommodation.

**Discussion:** The adapted Measuring the Quality of Prison Life questionnaire is a reliable and valid instrument for measuring quality of life in forensic psychiatric hospitals and can be used in the future to compare hospitals and identify the strengths and weaknesses of each.

## Introduction

Detaining patients in forensic psychiatric hospitals has two objectives, i.e., to treat mental illness and reduce the risk of relapse. A large proportion of forensic psychiatric patients spends many years in a closed forensic psychiatric hospital (mean duration for patients with severe mental disorders: 4.6 years, range: 0.5–16.1 years; Dessecker, 2008). In these institutions, the daily routine is firmly structured and the opportunities for independent action are very limited. These narrow framework conditions sometimes block individuating personality maturation and limit therapeutic options. Therefore, to enable patients to develop positively, we need to assess their quality of life and adapt the living conditions to their needs.

Quality of life describes people's well-being and satisfaction with their current living conditions (Lehmann, 1983). According to the World Health Organization Quality of Life Workgroup (WHOQOL), quality of life is defined as “an individual's perception of their position in life in the context of the culture and value systems in which they live and in relation to their goals, expectations, standards and concerns. It is a broad ranging concept affected in a complex way by the person's physical health, psychological state, personal beliefs, social relationships and their relationships to salient features of their environment” (Whoqol Group, 1995). Most authors consider quality of life as a multidimensional concept that includes both objective (e.g., health, income) and subjective indicators (e.g., satisfaction with social relations).

Experience from hospital practice shows, that patients evaluate their quality of life in forensic psychiatric hospitals quite differently. Some experience their detainment as unpleasant and negative, whereas others find the structured environment to be beneficial and protective. Given this potential range of subjective experiences, the present study aimed to develop and evaluate a questionnaire for mentally ill offenders that measures the quality of life in forensic psychiatric hospitals. The questionnaire “Measuring the Quality of Prison Life” (MQPL) by Liebling et al. (2011), which was designed for use in correctional facilities, served as a template. Liebling et al. used a bottom-up approach, i.e. they accompanied inmates in five different prisons for a year, had numerous conversations with them and thus gained insight into the issues that were relevant to them. From their observations at the grassroots level, they created a questionnaire with over 100 items, which was subsequently evaluated and validated in various prisoner populations (*N* = 1,147). In this way, Liebling's working group succeeded in identifying and statistically recording the parameters that were particularly important in the prisoners' daily lives. The questionnaire is now used worldwide and results are available from England, Spain, Norway, Sweden, Australia, Kosovo, and New Zealand (e.g., Leeson et al., 2015; Skar et al., 2019).

Liebling et al. found large differences between individual prisons with regard to prisoner's well-being and their current psychological distress (Liebling, 2009; Crewe et al., 2015). For example, some institutions are experienced as being more punitive than others. Significant correlations were also found between inmates' quality of life and the suicide rates in the respective institutions (Liebling et al., 2005). A study from Norway by Johnsen et al. (2011), examined the influence of prison size on inmates' evaluation of quality of life and found that they rated it most positively in prisons with fewer than 50 inmates (Johnsen et al., 2011). Skar et al. (2019) performed a study in a prison in Kosovo to investigate whether inmates' quality of life was associated with their mental health and the level of violence. They found a significant negative relationship between anxious symptoms, physical and psychological violence and quality of life (Skar et al., 2019).

Numerous studies have examined quality of life among prison inmates, and research on quality of life in forensic psychiatric hospitals is also making progress (Radoschewski, 2000; Nieuwenhuizen et al., 2002; Schalast et al., 2008; Vorstenbosch et al., 2014; Tonkin, 2016). However, only a small proportion of the studies focus on quality of life as a multidimensional construct that covers both objective living conditions and subjective well-being. Sampson et al. (2016) compared forensic psychiatric care in 18 European countries by conducting interviews with mental health experts. They concluded that improving the well-being and quality of life of long-term housed patients was essential for treatment (Sampson et al., 2016). Two studies from the Netherlands showed that patients' own assessment of quality of life and the way staff assess patients' quality of life diverge (Schel et al., 2015; de Vries et al., 2016) and Büsselmann et al. (2020) revealed that the social aspects of quality of life of forensic psychiatric patients are associated with suicidal thoughts, the severity of depressive symptoms and hopelessness.

The aims of the present study were threefold: First, the Measuring the Quality of Prison Life questionnaire (Liebling et al., 2011) should be translated into German and adapted to the living conditions of forensic psychiatric hospitals to assess the quality of life of forensic psychiatric patients, including adding items on patient-therapist relationships. Second, for the psychometric evaluation of our adapted questionnaire, a one-time survey was carried out at 13 forensic psychiatric hospitals. The reliability of the main scale and subscales should be determined using internal consistency and the construct validity should be tested by means of a confirmatory factor analysis. Third, to test of responsiveness, it should be investigated whether significant differences can be found between different forensic hospitals or between different patient groups (older vs. younger, patients with long and patients with short length of stay).

## Materials and Methods

### Sample

A total of 255 forensic psychiatric patients (25 women, 230 men) took part in the study; however the data of 25 patients were excluded from the analysis because too many values were missing. All patients were detained according to Section 63 (severe mental disorder, *n* = 81; 35%) or Section 64 (substance use disorder, *n* = 149; 65%) of the German penal code.

An overview of sociodemographic and forensic-psychiatric characteristics of the two subsamples of patients (i.e., those with severe mental disorders and those with substance use disorders) is shown in Table 1.

**Table 1:** Sociodemographic and forensic-psychiatric characteristics of the participants.

	**Severe mental disorder** **(*n* = 81)** **M (*SD*; Range)** **% (*n*)**	**Substance use disorder (*n* = 149)** ***M* (*SD*; Range)** **% (*n*)**
Age (years)^a^	40.2 (13.2; 19–79)	33.2 (9.0; 20–68)
**Graduation**^**b**^
No graduation	17 (21%)	18 (12%)
Graduation after 9 years	37 (46%)	81 (54%)
Graduation after 10 years	12 (15%)	39 (26%)
Graduation from high school	14 (18%)	6 (7%)
**Diagnosis**
Substance-related disorder	7 (9%)	141 (95%)
Schizophrenia	27 (33%)	0
Schizophrenia and addiction	9 (11%)	3 (2%)
Personality disorder	29 (36%)	1 (1%)
Other	9 (11%)	4 (3%)
**Index Offence**^**c**^
Violent offense	42 (53%)	50 (34%)
Sexual assault	23 (29%)	2 (1%)
Offense against property	2 (3%)	24 (16%)
Arson	5 (6%)	2 (1%)
Violation of the narcotic act	1 (1%)	70 (47%)
Traffic offense	6 (8%)	1 (1%)
Treatment duration (months)^d^	71.3 (85.0; 1–360)	12.7 (10.0; 0–56)

a *missing data: n = 2;*

b *missing data: n = 1;*

c *missing data: n = 2;*

d *missing data: n = 9. SD, standard deviation*.

### Assessment of Socio-Demographic, Hospital, and Legal Data

Patients were asked for the following information: gender, age, highest school leaving certificate, duration of actual detention, diagnosis, legal terms of detaining, index offense, and level of movement allowed.

### Measuring the Quality of Life

With kind permission of the authors, we translated the MQPL questionnaire (Liebling et al., 2011) into German. The original questionnaire consists of 128 items and covers both positive and negative living conditions of inmates. Because the MQPL questionnaire is tailored to the needs of prison inmates, the items related to therapeutic help and support were inappropriate for forensic psychiatric patients and were omitted. Instead, we drew on our own prior work, the Questionnaire for Investigating Therapeutic Alliance in Forensic Setting (FTBF; Vasic et al., 2015). The FTBF takes into account the formal and infrastructural characteristics of forensic psychiatric hospitals. We adopted items on the patient-therapist relationship and satisfaction with the therapeutic process. The adapted version, named aMQPL, consisted of 73 items, which were assigned to 14 subscales: entry in forensic psychiatry, relationship with fellow inmates, relationship with caregivers, relationship with therapists, family contact, respect, fairness, transparency of procedures and decisions, safety, quality of accommodation, therapeutic options/personal development, suicide prevention, drug consumption and treatment of foreign patients. The items were answered on a five-point-Likert scale (1 = totally disagree; 5 = totally agree). To evaluate the questionnaire, we calculated the mean score for the subscales and the total score. The higher the respective mean score was, the more positive patients rated the specific aspects of their quality of life (reflected by the subscales) or their overall quality of life (reflected by the total score).

### Procedure

From February to November 2018 we recruited *N* = 255 forensic patients in 13 out of 14 Bavarian (German) forensic hospitals. The patients were informed about the aim and procedure of the study and about the fact that neither participation nor non-participation would have any advantages or disadvantages with respect to their treatment. In addition, they were not offered either payment or other forms of compensation. Subsequently, they were asked to decide whether or not they were willing to participate in this study. If they agreed to participate, patients gave written informed consent and received a sheet with contact details of the research team. They were informed that they could withdraw their consent at any time. Thus, the study was performed in accordance with the criteria of the Declaration of Helsinki. Participants completed the questionnaires in small groups in a separate room on the ward, and a research assistant was available to help.

### Statistical Analysis

The data were analyzed with IBM SPSS Statistics for Windows Version 25 (Armonk, NY: IBM Corp.). Item characteristics were determined by means of item difficulties and item discriminations. Reliability was calculated via internal consistency analyses (Cronbachs' alpha). The factorial validity was examined with the help of a confirmatory factor analysis.

Analyses of variance were performed to test statistically significant differences between the 13 participating forensic psychiatric hospitals. The mean value of the respective aMQPL-subscale/total scale was used as the dependent variable; the independent variable was the affiliation to one of the 13 hospitals.

To test whether there are correlations between patients' age and quality of life, Spearman correlations were calculated separately for patients with a severe mental disorder and for patients with substance use disorders. To check if the duration of their accommodation (above and below the 50th percentile of the distribution of the mean duration) was associated with the assessment of quality of life, *t*-tests for independent groups were calculated for each aMQPL-subscale and the aMQPL-total scale.

## Results

### Psychometric Evaluation of the Questionnaire

For the interpretation of item characteristics and internal reliability, we followed the guidelines by Bühner (2011): The item difficulties (in percent) should cover as wide a range as possible (0–100), since extreme difficulties also allow differentiation in peripheral areas of the covered domains. The item discrimination index corresponds to the correlation coefficient between the item response i and the total scale score. The total scale value is calculated as the sum of all items without item i. Good item discrimination indices are greater than *r*_*i*(*t*−*i*)_ = 0.30. Cronbach's alpha is a measure of the internal consistency of the scale or subscale. It indicates how strongly the individual items are related to each other. Cronbach's alpha should assume a value greater than *r* = 0.65.

Nine items and the 3 related subscales were excluded due to insufficient internal consistencies (Cronbach's alpha: suicide prevention = 0.335; drug use = 0.308 and treatment of foreign patients = 0.013). The results of the evaluation of the 64 remaining items and 11 subscales can be found in Table 2. Item difficulties ranged between 33.8 and 64.6. The reliability of the total scale can be rated as excellent (Cronbach's alpha of the total score: *r* = 0.953).

**Table 2:** Item characteristics and reliabilities of the adapted measuring the quality of prison life questionnaire.

**Scale**	**α**		**Item**		**FL**	***r_***i*(*t*−*i*)**_***
Entry into forensic psychiatry	0.599		1	When I first came into this hospital I felt looked after.	0.693^*^	0.487
			2	During my first few days in this hospital, caregivers took a personal interest in me.	0.776^*^	0.489
			3	The induction process in this hospital helped me to know exactly what to expect in the daily routine and when it would happen.	0.385^*^	0.333
			4	I felt extremely alone during my first 3 days. (–)	0.320^*^	0.272
Relationship with fellow inpatients	0.678		5	Fellow inpatients are like friends to me.	0.825^*^	0.537
			6	I trust my fellow inpatients.	0.825^*^	0.603
			7	My fellow inpatients take advantage of me. (–)	0.333^*^	0.339
			8	I have no problems with the other patients.	0.378^*^	0.375
Relationship with caregivers	0.843		9	Caregivers help me, when I need support.	0.731^*^	0.634
			10	Caregivers trust me.	0.765^*^	0.696
			11	I trust the caregivers.	0.883^*^	0.789
			12	The relationship between the caregivers and patients is good.	0.670^*^	0.606
Relationship with therapists	0.860		13	I get on well with my therapist.	0.805^*^	0.763
			14	My therapist wants the best for me.	0.840^*^	0.765
			15	I like going to the individual sessions.	0.717^*^	0.635
			16	I am afraid of my therapist. (–)	0.449^*^	0.447
			17	I trust my therapist.	0.868^*^	0.777
			18	My therapist makes decisions I don't like. (–)	0.551^*^	0.519
			19	My therapist takes time for me when I have an important concern, even outside of individual sessions.	0.570^*^	0.504
Family contact	0.488		20	The staff at this hospital help me stay in touch with my family or friends.	0.820^*^	0.275
			21	In this hospital, I can be visited often enough.	0.503^*^	0.482
			22	The visiting time is too short. (–)	0.161^*^	0.194
Respect	0.827		23	I've been treated respectfully in this hospital.	0.767^*^	0.651
			24	The atmosphere in this hospital is nice and friendly.	0.668^*^	0.600
			25	My concerns are taken seriously at this hospital.	0.729^*^	0.664
			26	Some of the treatment in this hospital is humiliating. (–)	0.535^*^	0.517
			27	The staff are argumentative toward the patients. (–)	0.526^*^	0.526
			28	I have been treated with respect at this hospital.	0.808^*^	0.677
Fairness	0.817		29	In this hospital, all patients are treated equally.	0.870^*^	0.745
			30	The house rules apply to everyone; there are no exceptions.	0.721^*^	0.648
			31	My rights as defined by law are respected in this hospital.	0.624^*^	0.494
			32	In this hospital, everyone is punished for misconduct in the same way.	0.722^*^	0.682
			33	All patients' rooms are checked with the same frequency.	0.500^*^	0.482
Transparency of procedures and decisions	0.810		34	In this hospital, decisions are not explained. (–)	0.581^*^	0.536
			35	The rules that apply in this hospital have been explained to me.	0.594^*^	0.538
			36	I know exactly what is expected of me.	0.656^*^	0.582
			37	When important decisions are made about me, it is explained to me how they have been made.	0.759^*^	0.691
			38	I believe that I have no influence on the progress of my stay at the hospital. (–)	0.565^*^	0.492
			39	When important decisions are made about me, I am involved.	0.597^*^	0.538
			40	The procedures in the hospital are well-organized.	0.608^*^	0.476
Safety	0.800		41	The staff at this hospital make me feel safe.	0.784^*^	0.618
			42	The staff react quickly in case of unexpected incidents and emergencies.	0.424^*^	0.392
			43	Bullying among patients is not tolerated in this hospital.	0.684^*^	0.657
			44	Bullying of patients by staff is not tolerated in this hospital.	0.605^*^	0.548
			45	Patients are treated correctly in the crisis intervention room/isolation room.	0.579^*^	0.505
			46	I feel safe from being assaulted in this hospital.	0.683^*^	0.620
Quality of accommodation	0.788		47	My room is big enough.	0.499^*^	0.438
			48	My room is well-equipped.	0.669^*^	0.620
			49	The meals are good.	0.567^*^	0.493
			50	I have the opportunity to cook for myself.	0.270	0.226
			51	I have enough money at my personal dispotion.	0.587^*^	0.497
			52	The common rooms on the ward are clean and tidy.	0.429^*^	0.361
			53	There are enough games (ludo, table football, etc.).	0.533^*^	0.487
			54	The hospital offers enough opportunities to stay physically fit.	0.543^*^	0.475
			55	There are plenty of frequent activities (baking cookies, excursions, etc.).	0.547^*^	0.464
			56	I have adequate opportunities to take care of myself.	0.453^*^	0.405
			57	I have adequate opportunities to keep my room clean.	0.503^*^	0.472
Therapeutic options/Personal development	0.853		59	In this hospital, they help me avoid getting into conflict with the law after being released.	0.725^*^	0.638
			60	I am encouraged to confront my offenses.	0.769^*^	0.694
			61	I am encouraged to set goals and work toward them.	0.752^*^	0.660
			62	My time here in the hospital is a chance for me to change.	0.727^*^	0.709
			63	On the whole, I am just spending my time here instead of making use of it. (–)	0.616^*^	0.582
			64	I benefit from the therapies that are offered.	0.774^*^	0.714
			65	I regularly participate in the offered therapies.	0.410^*^	0.374

*α, Cronbach's alpha as indicator of internal consistency; FL, standardized factor loadings;*

* *p < 0.05, r_i(t−i)_, item discrimination index*.

Factor structure was tested by confirmatory factor analysis and is given [Chi^2^(1897) = 3442.143; *p* < 0.001; Bollen-Stine bootstrap-corrected *p* = 0.008; RMSEA = 0.067; 90% confidence interval: 0.064–0.071; for interpretation: good models have values RMSEA ≤ 0.08]. Significant standardized factor loadings are listed in Table 2.

### Test of Responsiveness: Differences Between Forensic Psychiatric Hospitals

**Figure 1:**
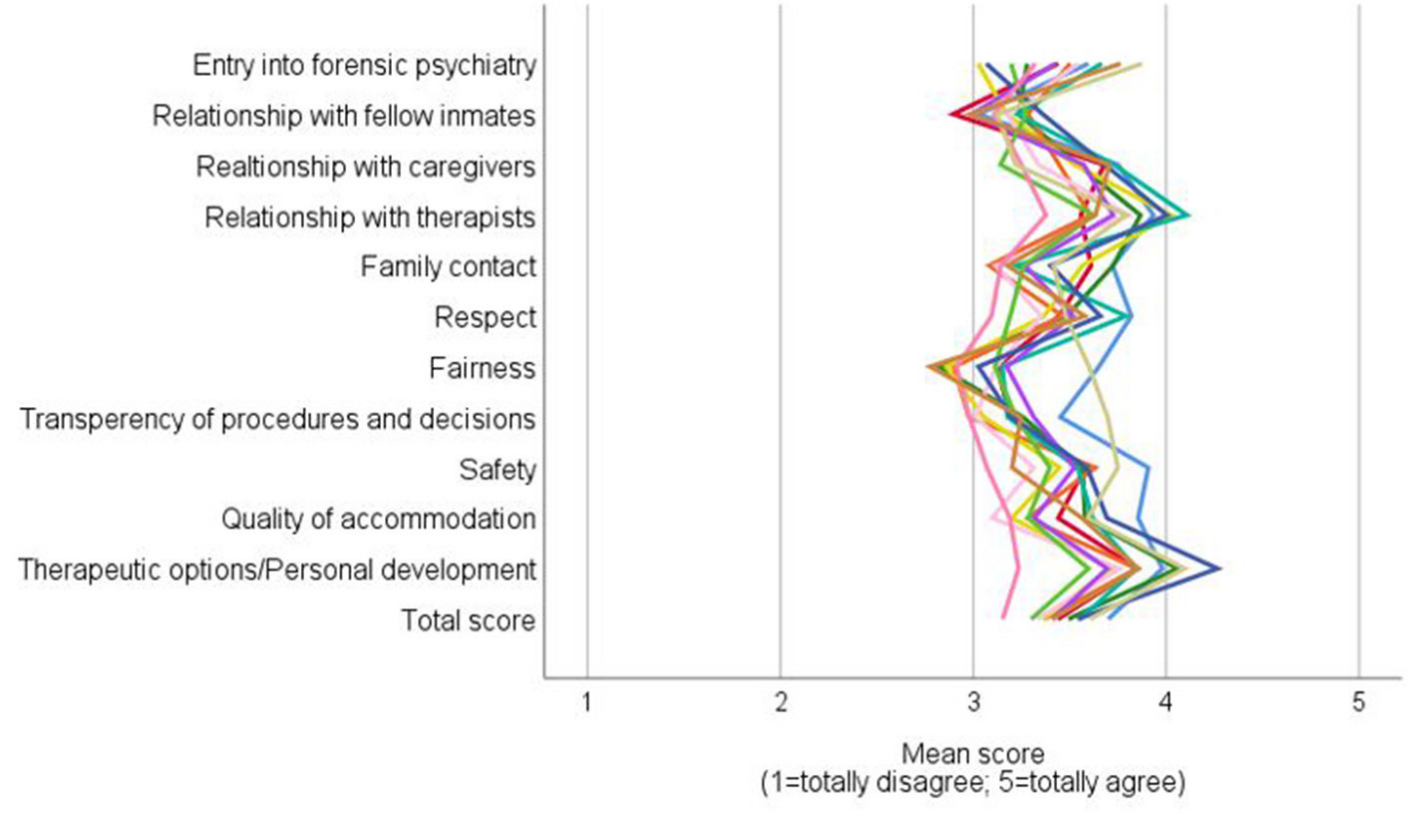
Mean values of the 13 participating Bavarian forensic psychiatric hospitals across the individual subscales and the total score of the aMOPL.

As can be seen in Figure 1, significant differences were found between the 13 participating forensic psychiatric hospitals in the following subscales: *e*ntry into forensic psychiatry [*F*_(12, 242)_ = 1.993; *p* = 0.026; part. Eta^2^ = 0.090], fairness [*F*_(12, 241)_ = 1.982; *p* = 0.026; part. Eta^2^ = 0.090], quality of accommodation [*F*_(12, 242)_ = 4.164; *p* < 0.001; part. Eta^2^ = 0.171] and therapeutic options/personal development [*F*_(12, 241)_ = 1.870; *p* = 0.039; part. Eta^2^ = 0.085].

### Test of Responsiveness: Associations Between Quality of Life and Patients' Age and Duration of Hospital Stay

For patients with severe mental disorders, the analyses further showed, that there was a significant negative correlation between the patients' age and the subscale family contact (Spearman's ρ = −0.222, *p* = 0.049). For patients with substance use disorders we found significant positive correlations between the patients' age and the subscales fairness (Spearman's ρ = 0.207, *p* = 0.011) and quality of accommodation (Spearman's ρ = 0.172, *p* = 0.036).

The duration of the hospital stay also influenced quality of life. Patients with severe mental disorders, who were accommodated in a forensic psychiatric hospital for 43 months or more rated entry in forensic psychiatry [*t*_(70)_ = −2.622; *p* = 0.011; *d*_*Cohen*_ = −0.627], relationship with caregivers [*t*_(70)_ = −2.107; *p* = 0.039; *d*_*Cohen*_ = −0.504], transparency of procedures and decisions [*t*_(70)_ = −3.034; *p* = 0.003; *d*_*Cohen*_ = −0.725] and therapeutic options/personal development [*t*_(70)_ = −2.257; *p* = 0.027; *d*_*Cohen*_ = −0.540] more positive than patients who were accommodated for a shorter period of time (< 43 months). Patients with substance use disorders who were accommodated for 12 or more months rated the quality of life more negatively than patients with a shorter length of stay, total score [*t*_(146)_ = 2.083; *p* = 0.039; *d*_*Cohen*_ = 0.345]. The same applied to the subscales relationships with therapists [*t*_(128, 838)_ = 2.301; *p* = 0.023; *d*_*Cohen*_ = 0.405], respect [*t*_(146)_ = 2.361; *p* = 0.020; *d*_*Cohen*_ = 0.391], and transparency of procedures and decisions [*t*_(146)_ = 3.153; *p* = 0.002; *d*_*Cohen*_ = 0.522].

## Discussion

This study aimed to analyze the psychometric properties of a translated and adapted version of the Measuring the Quality of Prison Life questionnaire (Liebling et al., 2011). The analysis of the psychometric properties of the adapted German questionnaire revealed good to excellent values for reliability and a confirmatory factor analysis confirmed the factor structure.

We found significant differences between the participating hospitals in the subscales entry into forensic psychiatry, fairness, quality of accommodation and therapeutic options/personal development. Because the aMQPL questionnaire allows the current quality of life at individual forensic psychiatric hospitals to be assessed, the aMQPL can be used in the future both to inform staff if any areas still need to be optimized and to compare conditions between hospitals.

Furthermore, our study shows that the age of patients in forensic psychiatric hospitals affects their quality of life. Young patients with substance use disorders feel treated more unfair than older patients. This finding gives rise to the question whether younger patients' concerns may be taken not so serious than older patients' concerns. In addition, young patients with substance use disorders rate the quality of accommodation significantly more negatively than older patients. One possible explanation for this difference may be that the living conditions and recreational opportunities available to young patients are not age-appropriate.

Patients with severe mental disorders rate their quality of life more positively the longer they have been detained in a forensic hospital (>3.5 years). The reason for that could be a therapeutically unintentional habituation to the forensic hospital. Being locked up for a long time creates helplessness, and over the years patients may become increasingly worried that they will not be able to cope with the practical demands of life outside the forensic hospital. Therapists and caregivers can try to reverse this effect of hospitalization by carefully preparing patients for discharge and relieving their worries about their new life “on the outside.”

In conclusion, a high quality of life should be ensured in forensic psychiatric hospitals to promote the best possible course of therapy. And the aMQPL appears to be a suitable self-assessment instrument for evaluating patients' quality of life. The developed questionnaire can be used with two different intentions: (a) to monitor the current status and further development of an individual forensic psychiatric hospital or (b) as an instrument to compare different forensic psychiatric hospitals with each other.

### Limitations

This study has some limitations. First, the sample consisted only German forensic psychiatric patients, so the results cannot be generalized to general psychiatric patients or forensic patients from other countries. Second, self-reported data can result in various biases.

## Data Availability Statement

The raw data supporting the conclusions of this article will be made available by the authors, without undue reservation.

## Ethics Statement

The studies involving human participants were reviewed and approved by Ethics Committee of the University Ulm, Germany. The patients/participants provided their written informed consent to participate in this study.

## Author Contributions

MD, JS, and MB designed the study. MB collected the data and wrote the initial draft of the manuscript. MB, JS, and ML analyzed the data. MB, JS, and LT interpreted the data. All authors had full access to all the data in the study and take responsibility for the integrity and accuracy of the data analysis, contributed to read, and approve the final version of the manuscript.

## Conflict of Interest

The authors declare that the research was conducted in the absence of any commercial or financial relationships that could be construed as a potential conflict of interest.
